# A genetic contribution from the Far East into Ashkenazi Jews via the ancient Silk Road

**DOI:** 10.1038/srep08377

**Published:** 2015-02-11

**Authors:** Jiao-Yang Tian, Hua-Wei Wang, Yu-Chun Li, Wen Zhang, Yong-Gang Yao, Jits van Straten, Martin B. Richards, Qing-Peng Kong

**Affiliations:** 1 State Key Laboratory of Genetic Resources and Evolution, Kunming Institute of Zoology, Chinese Academy of Sciences, Kunming 650223, China; 2 Key Laboratory of Animal Models and Human Disease Mechanisms, Kunming Institute of Zoology, Chinese Academy of Sciences, Kunming 650223, China; 3 KIZ/CUHK Joint Laboratory of Bioresources and Molecular Research in Common Diseases, Kunming 650223, China; 4 Kunming College of Life Science, University of Chinese Academy of Sciences, Beijing 100049, China; 5 Independent scholar, Bennekom, The Netherlands; 6 Department of Biological Sciences, School of Applied Sciences, University of Huddersfield, Queensgate, Huddersfield, HD1 3DH, UK

## Abstract

Contemporary Jews retain a genetic imprint from their Near Eastern ancestry, but obtained substantial genetic components from their neighboring populations during their history. Whether they received any genetic contribution from the Far East remains unknown, but frequent communication with the Chinese has been observed since the Silk Road period. To address this issue, mitochondrial DNA (mtDNA) variation from 55,595 Eurasians are analyzed. The existence of some eastern Eurasian haplotypes in eastern Ashkenazi Jews supports an East Asian genetic contribution, likely from Chinese. Further evidence indicates that this connection can be attributed to a gene flow event that occurred less than 1.4 kilo-years ago (kya), which falls within the time frame of the Silk Road scenario and fits well with historical records and archaeological discoveries. This observed genetic contribution from Chinese to Ashkenazi Jews demonstrates that the historical exchange between Ashkenazim and the Far East was not confined to the cultural sphere but also extended to an exchange of genes.

Consistent with their displaced ethno-history since the ancient Northern Kingdom of Israel was invaded and occupied by the Neo-Assyrian Empire^1^, contemporary Jews, including Ashkenazi Jews, Sephardic Jews, North African Jews and Middle Eastern Jews^2^, retain a genetic imprint of their Near Eastern ancestry, but have received a substantial contribution, to a variable extent, from their neighboring populations such as Europeans, Near Easterners, and North Africans^2^,^3^,^4^,^5^,^6^,^7^,^8^. However, it has hitherto remained unclear whether Jews received any genetic contribution from populations outside western Eurasia. Intriguingly, frequent communication has been observed between Jews and Chinese since the early centuries of the Common Era, plausibly initiated by the Silk Road. For instance, Hebrew letters and prayers in the 8^th^ century from ancient Jewish merchants were found in the northwestern region of China^9^. Some unearthed pottery figurines from the Tang Dynasty (618–907AD) have Semitic characteristics^9^, and synagogues were recorded in the epigraphy from the Ming (1368–1644AD) and Qing Dynasties (1644–1912AD)^9^,^10^. Nonetheless, such connections, as revealed by the archaeological discoveries and historical records, have been confined to economic and cultural exchanges; so far, no direct evidence of a genetic contribution from Chinese into Jews has been reported.

To address the issue of whether Jews received any genetic contribution from the Far East, and thus shed more light on their ethno-origins, mitochondrial DNA (mtDNA) variation (mainly from the control region of the molecule, plus some coding-region variants) of 1,930 Jews and 21,191 East Asians, retrieved from previous studies as well as our unpublished data, were considered and analyzed, with especial attention to pinpointing eastern Eurasian haplogroups in Jews (Supplementary Table S1). Then, mtDNA control region variants of an additional 32,474 Eurasian individuals were analyzed to gain further insights into the phylogeographic distribution of M33c (Supplementary Table S1), so that the total number of Eurasian mtDNAs considered here was 55,595. Our results do reveal a direct genetic connection, as manifested by the sharing of some Eastern Eurasian haplogroups e.g. N9a, A, and M33c, between Jews and Chinese. Further analyses, including phylogeny reconstruction with the aid of new mtDNA genomes, confirm that this connection was established at least by a founder lineage M33c2. The differentiation time of this lineage is estimated to ~1.4 kilo-years ago (kya), which fits well with the historical records and, most importantly, indicates that the exchange between Jews and the Far East was not confined to culture but also extended to the demic.

## Results and Discussion

Our analysis of the mtDNA variation in a total of 23,121 individuals from East Asian populations and Jews reveals that mtDNAs of four Ashkenazi Jewish individuals can be allocated into eastern Eurasian haplogroups A and N9a, suggesting that Ashkenazi Jews received a genetic contribution from East Asia (Table 1). Intriguingly, our results also disclose that 14 eastern Ashkenazi Jews belong to haplogroup M33c (Table 1), for which sister clusters, M33a, M33b and M33d, are prevalent in the Indian Subcontinent and thus most plausibly trace their origins there^11^,^12^.

**Figure 1:**
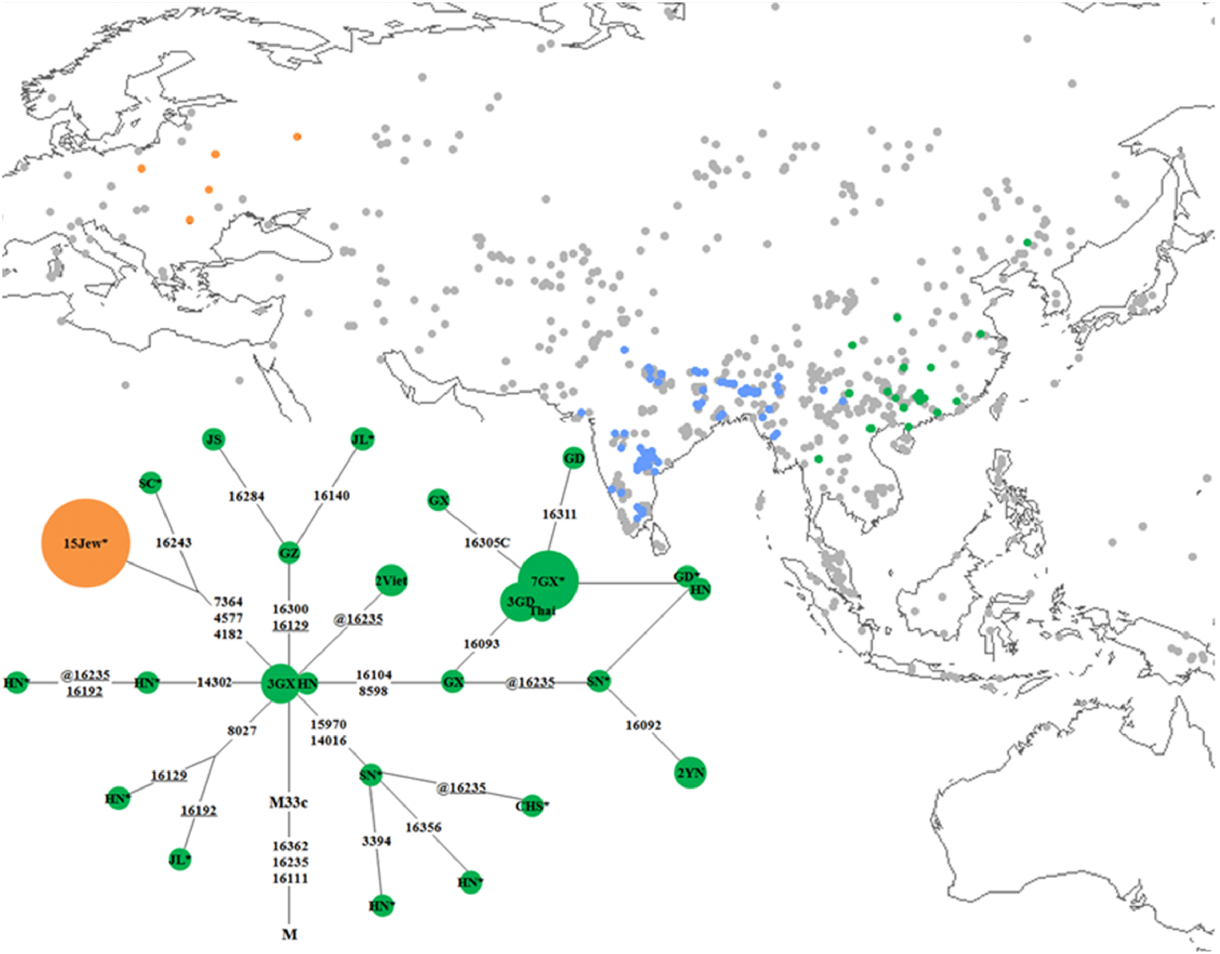
Median-joining network of haplogroup M33c. The median-joining network is reconstructed on the basis of mtDNA hypervariable segment I (HVS-I) variation. The sampling locations are shown by different colors in the map. Transversions are highlighted by adding suffixes “A”, “C”, “G”, and “T”. The prefix @ designates back mutation, whereas recurrent variants are underlined. * denotes that this individual's whole-mtDNA genome information is shown on the phylogenetic tree. The size of the circle is in proportion to the number of individuals. The geographic locations are abbreviated as follows: CHS (Hunan or Fujian), GD (Guangdong), GX (Guangxi), GZ (Guizhou), HN (Hunan), JL (Jilin), JS (Jiangsu), SC (Sichuan), SN (Shaanxi), Thai (Thailand), Viet (Vietnam), and YN (Yunnan). Note: 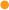
M33c individuals in Europe. 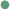
M33c individuals in Asia. 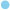
M33a, M33b or M33d individuals. 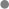
Sampling locations of all the other samples considered in this study. The map was created by the Kriging algorithm of the Surfer 8.0 package. More details regarding the populations are displayed in Supplementary Table S1.

To achieve further insight into the phylogeographic distribution of M33c, mtDNA variants (mainly from the control region) of an additional 32,474 Eurasian individuals were analyzed, so that the total number of Eurasian mtDNAs considered here was 55,595. As shown in Table 1, besides the 14 Ashkenazi Jewish M33c lineages, an additional 38 M33c mtDNAs (with the specific control-region motif showing transitions at positions 16111, 16223, 16235, and 16362) were pinpointed, among which 34 are from China, 2 from Vietnam, and 1 from Thailand, with the remaining individual most likely from Europe but with ambiguous ancestry. Thus, despite the restricted distribution of M33a, M33b and M33d in South Asia, it is most likely that M33c originated, or at least differentiated, in eastern Asia. This notion receives clear support from the median network, in which virtually all of the diversity of this haplogroup is observed in China (Figure 1).

**Figure 2:**
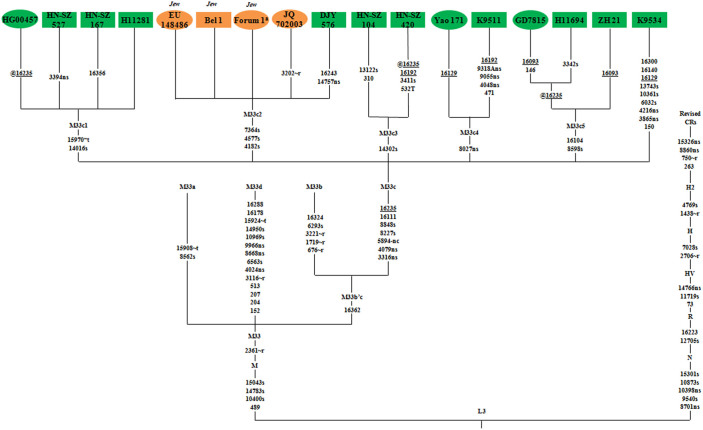
Phylogenetic tree of haplogroup M33c. The nucleotide positions in the sequences are scored relative to the rCRS^27^. Transversions are annotated by adding suffixes “A” and “T”. The recurrent variants are underlined and prefix @ designates a back mutational event; “s” means synonymous and “ns” means nonsynonymous mutation; “nc” refers to mutations at the intergenic noncoding regions in segments 577–16023; and “r” and “t” denote mutations in rRNA genes and tRNA genes, respectively. Length polymorphisms (e.g., 309.1C, 309.2C and 315.1C) are disregarded from the analysis. The newly sequenced samples in this study are marked in rectangles, while mtDNAs from the published literature are displayed in ellipses. Note: ^a^ This individual is from A Genetic Genealogy Community (http://eng.molgen.org/viewtopic.php?f=41&t=141).

To shed light on the phylogeny within haplogroup M33c, 11 mtDNAs, covering the widest range of internal variation within the haplogroup, were chosen for whole-mtDNA genome sequencing. In good agreement with the previous result^13^, the resulting phylogenetic tree (Figure 2), incorporating five previously reported mtDNA genomes^13^,^14^,^15^ as well as one whose information was released online (A Genetic Genealogy Community; http://eng.molgen.org), confirms that M33c is defined by mutations at positions 3316, 4079, 5894, 8227, 8848, 16111, and 16235. Of note is that five clades within M33c appear respectively characterized by diagnostic coding-region variant(s), and these are named M33c1 to M33c5 here. With the exception of M33c2, all the samples in these clades are from China. The likely origin of M33 in South Asia and the restriction to China of M33c, dating to 10 kya according to the estimation based on whole-mtDNA genome, implies some dispersal from South to East Asia in the immediate postglacial.

Intriguingly, sub-haplogroup M33c2 (defined by three additional coding-region variants at positions 4182, 4577, and 7364) consists of three different haplotypes (one seen in three Ashkenazi Jews, another in a single Chinese individual and the third in the likely European with unknown ethnicity). Although there is no control-region variant in the defining motif of M33c2, multiple lines of evidence suggest that the pinpointed 14 Ashkenazi Jewish M33c mtDNAs most likely all belong to this clade: (1) all of the 14 mtDNAs share an identical control-region motif (Table 1); (2) the three completely sequenced Ashkenazi Jewish mtDNAs with this motif (EU148486, Bel 1 and Forum 1) belong to M33c2 (Figure 2); (3) M33c shows a virtually exclusive distribution in Ashkenazi Jews in western Eurasia, even though 55,595 mtDNAs have been checked (Table 1 and Supplementary Table S1). Thus, it is plausible that the unknown European individual (JQ702003) was in fact from a Jewish population or had Ashkenazi Jewish ancestry.

Age estimates for M33c2 are similar whether based either on the whole genome or on the control region alone (Table 2), and the age of ~1.4 kya fits well with the medieval operation of the Silk Road. We note that this is an upper bound for the gene flow event during which the lineage was assimilated into the Ashkenazim; it is the age of the subclade overall, which most likely arose within China, and indeed there is no variation at all within the Jewish lineages, suggesting a very recent event. If we assume that the unidentified European lineage belongs within the Ashkenazi diversity, we can date the Ashkenazi subclade itself more specifically to about 640 years ago – around 1350AD. This in turn would then provide a minimum point estimate for the age of the gene flow event (although the range taking account of errors in the estimates is of course much wider).

The ancient Silk Road was an important transportation hub connecting China and the Mediterranean region from the Han Dynasty (206BC–220AD) onwards, and there are likely to have been Jewish merchants at the eastern end of the Silk Road from the early centuries AD. Moreover, Jewish merchants in Europe, referred to as Radhanites, were involved in trade between west and east as early as the ninth century^16^. It has been suggested, on the basis of contrasts between patterns of mtDNA and Y-chromosome variation^17^, that such merchants may have formed the nucleus for a number of extant Jewish communities.

Ashkenazi origins are controversial^18^. According to recent archaeological evidence, the Jewish community of Cologne, mentioned by Emperor Constantine in 321AD, existed in the city continuously until they had to leave in 1423–1424AD^19^. This suggests that Ashkenazi Jewry may date to Roman times, possibly originating in Italy, which is also suggested by analysis of mtDNA^8^ and autosomal data^20^. An early eastern European Ashkenazi origin from Italy (first millennium and earlier) would also agree with the finding that an origin mainly from Germany^21^ or another central or western European country^18^ during the late Middle Ages, is demographically not possible. Recent work also suggests a sizable Jewish presence in eastern Germany (the Danube region, rather than the Rhineland) prior to the expansion in Poland between 1500 and 1650AD^22^. The M33c2 mtDNAs are confined to eastern European Ashkenazim in the present database (the single unknown example is of likely East European ancestry^14^), suggesting that these groups had contacts to the east to the extent that they mediated female gene flow.

Extensive genetic admixture has been observed in populations residing around the ancient Silk Road region^23^,^24^. Our currently observed genetic imprint echoes the previously observed ancient communications between Jews and Chinese and, most significantly, implies that such historical exchanges were not confined to the cultural realm but involved gene flow. This unexpected ancient genetic connection between Ashkenazi Jews and the Far East, as witnessed at least by mtDNA haplogroup M33c2, provides the first evidence for a significant genetic contribution from Chinese to eastern European Ashkenazi Jews that was most likely mediated by the Silk Road between around 640 and 1400 years ago. Although the involvement of male Jewish traders has been suggested before^17^, our results, focusing on the female line of descent, specifically point to the involvement also of women. Well-resolved evidence from the male-specific part of the Y chromosome and from the autosomes would help to further illustrate the rather complex, pan-Eurasian ethno-history of Jews.

## Methods

### mtDNA Data collection and mining

mtDNA variation (mainly from control region) of 23,121 East Asians and Jews, retrieved from previous studies as well as our unpublished data, were considered and analyzed, with especial attention to pinpointing the eastern Eurasian haplogroups in Jews. Then, additional 32,474 individuals were analyzed to gain further insights into the phylogeographic distribution of M33c, leading the total number of Eurasian mtDNAs considered here to 55,595. The study project was approved by the Ethics Committee at Kunming Institute of Zoology, Chinese Academy of Sciences. Each participant was informed about the study and provided informed consent. All mtDNAs collected and considered in the present study were first allocated to haplogroups, based mainly on their control-region motifs, which were then further confirmed by typing specific coding-region variation according to the PhyloTree (mtDNA tree Build 16^25^; http://www.phylotree.org/).

### DNA amplification and sequencing

For haplogroups of interest, special attention was paid to the intrinsic phylogeny reconstructed on entire mitogenome information. In this way, entire mitogenomes for 11 selected representatives from haplogroup M33c were amplified, sequenced, and dealt with as described elsewhere^13^,^26^. The sequencing outputs were edited and aligned by Lasergene (DNAStar Inc., Madison, Wisconsin, USA) and compared with the revised Cambridge Reference Sequence (rCRS)^27^.

### Data analysis

The median-joining network of M33c was constructed manually^28^ and then confirmed using Network 4.612 (http://www.fluxus-engineering.com/sharenet.htm). The most parsimonious phylogenetic tree (Figure 2) was reconstructed by hand as carried out previously^13^,^26^. The coalescence ages were estimated by the ρ ± σ method^29^,^30^ and maximum likelihood (ML) analysis. Recently corrected calibrated mutation rates^31^ were adopted in the ρ statistic and the ML analysis.

## Author Contributions

Q.-P.K. designed the research; Y.-C.L., W.Z. and Y.-G.Y. collected the samples; J.-Y.T., H.-W.W. and Y.-C.L. collected the data; J.-Y.T. and H.-W.W. performed the experiments; J.-Y.T., H.-W.W., Y.-C.L. and Q.-P.K. analyzed data; J.-Y.T., Y.-C.L., J.v.S., M.B.R. and Q.-P.K. wrote the paper.

## Supplementary Material

Supplementary Information Sample information of populations analyzed in present study

## Figures and Tables

**Table 1:** The shared eastern Eurasian haplotypes between Ashkenazi Jews and Chinese

Sample ID	Haplogroup	HVS-I (16000+)	HVS-II	Population	Region/Country	Reference
AS2C5	A	182C 183C 189 193.1C 223 290 319 362	73 150 152 204 235 263	Ashkenazi	Hungary	32
472	N9a	129 223 257A 261	73 146 150 263	Ashkenazi	Russia	7
75916	N9a	129 223 257A 261	73 146 150 263 309.1C 309.2C 315.1C	Ashkenazi	Belarus	Family Tree DNA
110612	N9a	129 223 257A 261	73 146 150 263 309.1C 309.2C 315.1C	Ashkenazi	Belarus	Family Tree DNA
Bel 1^a^	M33c	111 223 235 362 (519)	73 263 315.1C (489)	Ashkenazi	Belarus	This study
EU148486^a^	M33c	111 223 235 362 (519)	73 263 315.1C (489)	Ashkenazi	Belarus	Family Tree DNA
4130^b^	M33c	111 223 235 362 (519)	73 263 315.1C (489)	Ashkenazi	Belarus	Family Tree DNA
135479^b^	M33c	111 223 235 362 (519)	73 263 315.1C (489)	Ashkenazi	Western Ukraine	Family Tree DNA
266609^b^	M33c	111 223 235 362 (519)	73 263 315.1C (489)	Ashkenazi	Western Ukraine	Family Tree DNA
N50366^b^	M33c	111 223 235 362 (519)	73 263 315.1C (489)	Ashkenazi	Western Ukraine	Family Tree DNA
N105293^b^	M33c	111 223 235 362 (519)	73 263 315.1C (489)	Ashkenazi	Russia	Family Tree DNA
50126^b^	M33c	111 223 235 362 (519)	73 263 315.1C (489)	Ashkenazi	Russia	Family Tree DNA
289247	M33c	111 223 235 362 (519)		Ashkenazi	Western Ukraine	Family Tree DNA
403	M33c	111 223 235 362 (519)	73 263	Ashkenazi	Belarus	7
404	M33c	111 223 235 362 (519)	73 263	Ashkenazi	Poland	7
405	M33c	111 223 235 362 (519)	73 263	Ashkenazi	Poland	7
406	M33c	111 223 235 362 (519)	73 263	Ashkenazi	Romania	7
Forum 1a,c	M33c	111 223 235 362 (519)	73 263 315.1C (489)	Jew	Belarus/Russia/Lithuania	A Genetic Genealogy Community
JQ702003^a^	M33c	111 223 235 362 (519)	73 263 315.1C (489)	N.A.	N.A.	14
HN-SZ420^a^	M33c	111 192 223 362 (519)	73 263 315.1C (489)	Han	Hunan, China	33, This study
HN-SZ167^a^	M33c	111 223 235 356 362 (519)	73 263 309.1C 315.1C (489)	Han	Hunan, China	33, This study
HN-SZ104^a^	M33c	111 223 235 362 (519)	73 263 308-310d 315.1C (489)	Han	Hunan, China	33, This study
HN-SZ527^a^	M33c	111 223 235 362 (519)	73 263 309.1C 315.1C (489)	Han	Hunan, China	33, This study
JSH08058	M33c	111 129 223 235 284 300 362 (519)	73 150 263 315.1C	Han	Jiangsu, China	This study
K9534^a^	M33c	111 129 140 223 235 300 362 (519)	73 150 263 309.1C 315.1C (489)	Han	Jilin, China	This study
K9511^a^	M33c	111 192 223 235 362 (519)	73 263 315.1C 471 (489)	Han	Jilin, China	This study
H11694^a^	M33c	104 111 223 362 (519)	73 263	Han	Shaanxi, China	This study
H11281^a^	M33c	111 223 235 362 (519)	73 263 309.1C 315.1C	Han	Shaanxi, China	This study
DJY576^a^	M33c	111 223 235 243 362 (519)	73 263 309.1C 315.1C	Han	Sichuan, China	This study
Zhuang21^a^	M33c	093 104 111 223 235 362 (519)	73 263 309.1C 315.1C (489)	Zhuang	Guangxi, China	This study
Zhuang74	M33c	093 104 111 223 235 362 (519)	73 263 309.1C 315.1C (489)	Zhuang	Guangxi, China	This study
Zhuang9	M33c	093 104 111 223 235 362 (519)	73 263 315.1C (489)	Zhuang	Guangxi, China	This study
HN-SZ416	M33c	093 104 111 223 362 (519)	73 263 309.1C 315.1C (489)	Han	Hunan, China	33
Dongguan-65	M33c	093 104 111 223 235 311 362	73 263 210 309.1C 310 315.1C	Han	Guangdong, China	34
539	M33c	093 104 111 223 235 362		Han	Guangxi, China	35
129	M33c	093 104 111 223 235 362		Han	Guangxi, China	35
136	M33c	093 104 111 223 235 362		Han	Guangxi, China	35
499	M33c	093 104 111 223 235 362		Han	Guangxi, China	35
370	M33c	104 111 223 235 362		Han	Guangxi, China	35
149	M33c	111 223 235 362		Han	Guangxi, China	35
Viet0121	M33c	111 223 362 (519)	73 203 263 309.1C 309.2C 315.1C (489)	Vietnamese	Vietnam	36
GD7815^a^	M33c	093 104 111 223 362	73 146 263 309.1C 315.1C	Han	Guangdong, China	13
Yao171^a^	M33c	111 129 223 235 362 (519)	73 263 309.1C 309.2C 315.1C	Yao	Hunan, China	13
MK13	M33c	111 129 223 235 300 362		Kam-Tai	Guizhou, China	37
Kinh117	M33c	111 223 362 (519)		Kinh	Vietnam	38
(03B)045	M33c	093 104 111 223 235 362 (519)	73 263 309.1C 315.1C	Han	Guangdong, China	39
YZ-Tib05-23	M33c	092 104 111 223 362		Tibetan	Yunnan, China	40
YZ-Tib05-8	M33c	092 104 111 223 362		Tibetan	Yunnan, China	40
MHN43	M33c	111 223 235 362		Miao	Hunan, China	41
YBP28	M33c	093 104 111 223 235 362		Yao	Guangdong, China	41
YLO22	M33c	093 104 111 223 235 305C 362		Yao	Guangxi, China	41
YLO03	M33c	111 223 235 362		Yao	Guangxi, China	41
YLO23	M33c	111 223 235 362		Yao	Guangxi, China	41
GD7821	M33c	093 104 111 223 235 362	73 263 309.1C 315.1C	Han	Guangdong, China	42
HG00457^a^	M33c	111 223 362 (519)		Han	Hunan/Fujian, China	15
TL397	M33c	093 101 111 223 235 362 (519)		Thai	Thailand	43

^a^The whole-mtDNA genome information is displayed on the phylogenetic tree (Figure 2).

^b^The individual has coding region information confirming its haplogroup assignment from the Family Tree DNA (www.familytreedna.com).

^c^This sequence is from A Genetic Genealogy Community (http://eng.molgen.org/viewtopic.php?f=41&t=141).

N.A. = not available.

All the samples in this study have confirmed G2361A mutation when assigning their haplogroup. The mutations (e.g. 489 and 16519) outside HVS-I and HVS-II are listed in the parentheses. The haplogroups A and N9a mtDNAs in the Far East are not shown here.

**Table 2:** Ages of the major clades of haplogroup M33c estimated from control-region and whole-mtDNA genome data with 95% confidence intervals

	Control region			Whole-mtDNA genome			
	Rho			Rho			Maximum Likelihood
Haplogroup	*n*	ρ ± σ	Age (kya)	*n*	ρ ± σ	Age (kya)	Age (kya)
M33c	52	1.13 ± 0.47	21.38 [4.1;38.7]	17	3.88 ± 0.76	10.29 [6.2;14.4]	8.95 [5.2;12.8]
M33c2	16	0.06 ± 0.06	1.18 [0;3.5]	5	0.60 ± 0.35	1.55 [0;3.3]	1.55 [0;3.3]
M33c2*	–	–	–	4	0.25 ± 0.25	0.64 [0;1.9]	0.64 [0;1.9]

M33c2*: the Chinese individual is not considered for the estimation.

## References

[b1] Josephus F. The Antiquities of the Jews (Echo Library, 2006).

[b2] Ostrer H. A genetic profile of contemporary Jewish populations. Nat. Rev. Genet. 2, 891–898 (2001). 11715044 10.1038/35098506

[b3] Behar D. M. *et al.* The genome-wide structure of the Jewish people. Nature 466, 238–242 (2010). 20531471 10.1038/nature09103

[b4] Atzmon G. *et al.* Abraham's children in the genome era: major Jewish diaspora populations comprise distinct genetic clusters with shared Middle Eastern Ancestry. Am. J. Hum. Genet. 86, 850–859 (2010). 20560205 10.1016/j.ajhg.2010.04.015 PMC3032072

[b5] Kopelman N. *et al.* Genomic microsatellites identify shared Jewish ancestry intermediate between Middle Eastern and European populations. BMC Genet. 10, 80 (2009). 19995433 10.1186/1471-2156-10-80 PMC2797531

[b6] Need A. C., Kasperavičiūtė D., Cirulli E. T. & Goldstein D. B. A genome-wide genetic signature of Jewish ancestry perfectly separates individuals with and without full Jewish ancestry in a large random sample of European Americans. Genome Biol. 10, R7 (2009). 19161619 10.1186/gb-2009-10-1-r7 PMC2687795

[b7] Behar D. M. *et al.* The matrilineal ancestry of Ashkenazi Jewry: portrait of a recent founder event. Am. J. Hum. Genet. 78, 487–497 (2006). 16404693 10.1086/500307 PMC1380291

[b8] Costa M. D. *et al.* A substantial prehistoric European ancestry amongst Ashkenazi maternal lineages. Nat. Commun. 4, 2543 (2013). 24104924 10.1038/ncomms3543 PMC3806353

[b9] Pan G. The Jews in China (China Intercontinental Press, 2007).

[b10] Shapiro S. Jews in old China: Studies by Chinese Scholars (Hippocrene Books, 2001).

[b11] Sun C. *et al.* The dazzling array of basal branches in the mtDNA macrohaplogroup M from India as inferred from complete genomes. Mol. Biol. Evol. 23, 683–690 (2006). 16361303 10.1093/molbev/msj078

[b12] Chandrasekar A. *et al.* Updating phylogeny of mitochondrial DNA macrohaplogroup m in India: dispersal of modern human in South Asian corridor. PLoS ONE 4, e7447 (2009). 19823670 10.1371/journal.pone.0007447 PMC2757894

[b13] Kong Q. P. *et al.* Large-scale mtDNA screening reveals a surprising matrilineal complexity in East Asia and its implications to the peopling of the region. Mol. Biol. Evol. 28, 513–522 (2011). 20713468 10.1093/molbev/msq219

[b14] Behar D. M. *et al.* A “Copernican” reassessment of the human mitochondrial DNA tree from its root. Am. J. Hum. Genet. 90, 675–684 (2012). 22482806 10.1016/j.ajhg.2012.03.002 PMC3322232

[b15] Zheng H. X. *et al.* Major population expansion of East Asians began before Neolithic Time: Evidence of mtDNA genomes. PLoS ONE 6, e25835 (2011). 21998705 10.1371/journal.pone.0025835 PMC3188578

[b16] McCormick M. Origins of the European Economy: Communications and Commerce AD 300–900 688–693 (Cambridge University Press, 2002).

[b17] Goldstein D. B. Jacob's Legacy: A Genetic View of Jewish History. (Yale University Press, 2008).

[b18] van Straten J. The Origin of Ashkenazi Jewry: The Controversy Unraveled. (New York: Walter de Gruyter & Co, 2011).

[b19] Schütte S. & Gechter M. Köln: archäologische zone Jüdisches Museum: von der Ausgrabung zum Museum-Kölner Archäologie zwischen Rathaus und Praetorium: Ergebnisse und Materialien 2006–2011. (Jüdisches Museum, 2011).

[b20] Behar D. M. *et al.* No evidence fom genome-wide data of a Khazar origin for the Ashekanzi Jews. (Hum. Biol., in press) (2013). 10.3378/027.085.0604 25079123

[b21] van Straten J. Early modern Polish Jewry: The Rhineland hypothesis revisited. Hist. Method. 40, 39–50 (2007).

[b22] King R. D. Migration and linguistics as illustrated by Yiddish. In Reconstructing languages and cultures, Polomé P. C., & Winter W., eds. ed. 419–439 (Berlin/New York: Mouton de Gruyter, 1992).

[b23] Comas D. *et al.* Trading genes along the silk road: mtDNA sequences and the origin of central Asian populations. Am. J. Hum. Genet. 63, 1824–1838 (1998). 9837835 10.1086/302133 PMC1377654

[b24] Yao Y. G., Kong Q. P., Wang C. Y., Zhu C. L. & Zhang Y. P. Different matrilineal contributions to genetic structure of ethnic groups in the Silk Road region in China. Mol. Biol. Evol. 21, 2265–2280 (2004). 15317881 10.1093/molbev/msh238

[b25] van Oven M. & Kayser M. Updated comprehensive phylogenetic tree of global human mitochondrial DNA variation. Hum. Mutat. 30, E386–E394 (2009). 18853457 10.1002/humu.20921

[b26] Kong Q. P. *et al.* Updating the East Asian mtDNA phylogeny: a prerequisite for the identification of pathogenic mutations. Hum. Mol. Genet. 15, 2076–2086 (2006). 16714301 10.1093/hmg/ddl130

[b27] Andrews R. M. *et al.* Reanalysis and revision of the Cambridge reference sequence for human mitochondrial DNA. Nat. Genet. 23, 147–147 (1999). 10508508 10.1038/13779

[b28] Bandelt H.-J., Macaulay V. & Richards M. Median networks: speedy construction and greedy reduction, one simulation, and two case studies from human mtDNA. Mol. Phylogenet. Evol. 16, 8–28 (2000). 10877936 10.1006/mpev.2000.0792

[b29] Forster P., Harding R., Torroni A. & Bandelt H.-J. Origin and evolution of Native American mtDNA variation: a reappraisal. Am. J. Hum. Genet. 59, 935 (1996). 8808611 PMC1914796

[b30] Saillard J., Forster P., Lynnerup N., Bandelt H.-J. & Nørby S. mtDNA variation among Greenland Eskimos: the edge of the Beringian expansion. Am. J. Hum. Genet. 67, 718–726 (2000). 10924403 10.1086/303038 PMC1287530

[b31] Soares P. *et al.* Correcting for purifying selection: an improved human mitochondrial molecular clock. Am. J. Hum. Genet. 84, 740–759 (2009). 19500773 10.1016/j.ajhg.2009.05.001 PMC2694979

[b32] Brandstätter A. *et al.* Mitochondrial DNA control region variation in Ashkenazi Jews from Hungary. Forensic Sci Int Genet 2, e4–e6 (2008). 19083780 10.1016/j.fsigen.2007.07.006

[b33] Zhang W. *et al.* A matrilineal genetic legacy from the Last Glacial Maximum confers susceptibility to schizophrenia in Han Chinese. J. Genet. Genomics 41, 397–407 (2014). 25064678 10.1016/j.jgg.2014.05.004

[b34] Chen F. *et al.* Analysis of mitochondrial DNA polymorphisms in Guangdong Han Chinese. Forensic Sci Int Genet 2, 150–153 (2008). 19083810 10.1016/j.fsigen.2007.10.122

[b35] Gan R. J. *et al.* Pinghua population as an exception of Han Chinese's coherent genetic structure. J. Hum. Genet. 53, 303–313 (2008). 18270655 10.1007/s10038-008-0250-x

[b36] Irwin J. A. *et al.* Mitochondrial control region sequences from a Vietnamese population sample. Int. J. Legal Med. 122, 257–259 (2008). 17960413 10.1007/s00414-007-0205-3

[b37] Li H. *et al.* Mitochondrial DNA diversity and population differentiation in southern East Asia. Am. J. Phys. Anthropol. 134, 481–488 (2007). 17668442 10.1002/ajpa.20690

[b38] Peng M. S. *et al.* Tracing the Austronesian footprint in Mainland Southeast Asia: a perspective from mitochondrial DNA. Mol. Biol. Evol. 27, 2417–2430 (2010). 20513740 10.1093/molbev/msq131

[b39] Wang W. Z. *et al.* Tracing the origins of Hakka and Chaoshanese by mitochondrial DNA analysis. Am. J. Phys. Anthropol. 141, 124–130 (2010). 19591216 10.1002/ajpa.21124

[b40] Wen B. *et al.* Analyses of genetic structure of Tibeto-Burman populations reveals sex-biased admixture in southern Tibeto-Burmans. Am. J. Hum. Genet. 74, 856–865 (2004). 15042512 10.1086/386292 PMC1181980

[b41] Wen B. *et al.* Genetic structure of Hmong-Mien speaking populations in East Asia as revealed by mtDNA lineages. Mol. Biol. Evol. 22, 725–734 (2005). 15548747 10.1093/molbev/msi055

[b42] Yao Y. G., Kong Q. P., Bandelt H.-J., Kivisild T. & Zhang Y.-P. Phylogeographic differentiation of mitochondrial DNA in Han Chinese. Am. J. Hum. Genet. 70, 635–651 (2002). 11836649 10.1086/338999 PMC384943

[b43] Kampuansai J. *et al.* Mitochondrial DNA Variation of Tai Speaking Peoples in Northern Thailand. ScienceAsia 33, 443–448 (2007).

