# Supplementary material for: A genetic contribution from the Far East into Ashkenazi Jews via the ancient Silk Road
Source: Sci Rep. 2015 Feb 11;5:8377. doi: 10.1038/srep08377 (PMC4323646; doi:10.1038/srep08377)
Supplement: Supplementary Information — Sample information of populations analyzed in present study [file srep08377-s1.pdf]

Supplementary Information

A genetic contribution from the Far East into Ashkenazi Jews via the ancient Silk Road

Jiao-Yang Tian, Hua-Wei Wang, Yu-Chun Li, Wen Zhang, Yong-Gang Yao, Jits van Straten, Martin B. Richards and Qing-Peng Kong*

* Correspondence author: Dr. Qing-Peng Kong

State Key Laboratory of Genetic Resources and Evolution, Kunming Institute of Zoology, Chinese Academy of Sciences, Kunming 650223, China.

Tel/Fax: +86-871-65197967; Email: kongqp@mail.kiz.ac.cn

Supplementary Table S1 Sample information of populations analyzed in present study (A total of 853 populations, 55,595 individuals)

Population, Region/Country	Sample Size	References
Jew		
Ethiopian Jewish, Ethiopia	41	1
Yemenite Jewish, Yemen	45	1
Ashkenazi, Austria/Hungary	29	2
Ashkenazi, Byelorussia	10	2
Ashkenazi, Czech Republic	15	2
Ashkenazi, Estonia	1	2
Ashkenazi, France	49	2
Ashkenazi, Germany	32	2
Ashkenazi, Latvia	8	2
Ashkenazi, Lithuania	30	2
Ashkenazi, Moldavia	1	2
Ashkenazi, Netherlands	21	2
Ashkenazi, Poland	170	2
Ashkenazi, Romania	97	2
Ashkenazi, Russia	63	2
Ashkenazi, Switzerland	3	2
Ashkenazi, Ukraine	54	2
Azerbaijan Mountain Jews, Azerbaijan	58	3
Belmonte Jews, Belmonte	30	3
Spanish Exilers, Bulgaria	71	3
Cochin Jews, India	45	3
Ethiopia Jews, Ethiopia	29	3
Mountain Jews, Georgia	74	3
Bnei Israel, India	34	3
Near East Jews, Iran	82	3
Near East Jews, Iraq	135	3
North Africa Jews, Libya	83	3
North Africa Jews, Morocco	149	3
North Africa Jews, Tunisia	37	3
Spanish Exilers, Turkey	123	3
Yemen Jews, Yemen	119	3
Spanish Exilers, Former Yugoslavia	1	3
Spanish Exilers, Greece	3	3
Spanish Exilers, Italy	9	3
Spanish Exilers, Netherlands	1	3
Spanish Exilers, Rodus	1	3
Spanish Exilers, Spain	3	3
Spanish Exilers, Surinam	1	3
Ashkenazi Jews, Hungary	173	4

East Asia		
Manchurian, China	40	5
Korean-Chinese, South Korea	51	5
Korean, South Korea	185	5
Chinese Han, Beijing, China	40	5
Hong Kong people, Hong Kong, China	377	6
Ancient workers, China	19	7
Japanese, Japan	4	8
Chinese, China	5	8
Tibetan, China	168	9
Tibetan, China	220	9
Tibetan, China	71	9
Tibetan, China	62	9
Tibetan, China	61	10
Tibetan, China	55	10
Tibetan, China	59	10
Tibetan, China	58	10
Tibetan, China	46	10
Monba, China	51	10
Tibetan, China	53	10
Lhoba, China	20	10
Tibetan, China	56	10
Tibetan, China	59	10
Tibetan, China	44	10
Tibetan, China	40	11
Bai, China	40	12
Hani, China	33	12
Lahu, China	15	12
Naxi, China	45	12
Pumi, China	35	12
Tibetan, China	35	12
Yi, China	56	12
Lahu, China	35	13
Lahu, China	32	14
Tibetan, China	24	14
Va, China	36	13
Va, China	22	14
Hui, China	45	15
Kazak, China	53	15
Mongoloid, China	49	15
Uygur, China	47	15
Uzbek, China	58	15
Bonan, China	95	16
Dongxiang, China	96	17

Salar, China	99	18
Salar, China	10	19
Yugur, China	100	20
Dongxiang, China	10	19
Xiaohe cemetery, China	20	21
Xiaohe cemetery , China	17	22
Daheyan, Xinjiang, China	58	23
Niya Site, Xinjiang, China	14	24
Yuansha Ruins, Xinjiang, China	15	24
Zagunluke Cemetery, Xinjiang, China	13	24
Ancient samples from Xinjiang, China	11	25
Taiyuan, Shanxi, China	2	22
Daur, China	45	26
Ewenki, China	47	26
Kor, China	48	26
Mongoloid, China	48	26
Oro, China	44	26
Kor-S, China	55	27
H-Anhui, China	42	28
H-Fujian, China	51	28
H-Gansu, China	45	28
H-Guangxi, China	26	28
H-Hunan, China	16	28
H-Jiangsu, China	67	28
H-Jiangxi, China	23	28
H-Liaoning, China	51	28
H-Neimeng, China	45	28
H-Qinhai, China	44	28
H-Shanghai, China	56	28
H-Shannxi, China	53	28
H-Sichuan, China	70	28
H-Yunnan, China	58	28
H-Zhejiang, China	61	28
H-GD, China	30	29
H-LN, China	51	29
H-QD, China	50	29
H-WH, China	42	29
H-XJ, China	47	29
H-YN, China	43	29
H-SD, China	76	30
Han, Yan Bian, Jilin, China	51	27
Ab-Taiwanese, China	180	31
TW-Han, China	155	32
H-GD, China	69	33

C-SH, China	120	34
H-CS, China	82	35
H-Xi'an, China	85	35
Aini, China	47	12
BAI, China	19	12
Jino, China	18	12
QH-Tib, China	56	12
Tujia, China	66	12
Tujia, China	31	12
YN-Dai, China	21	14
YN-Dai, China	38	11
MHN, China	103	36
MYN, China	39	36
YBN, China	19	36
YBP, China	35	36
YDB, China	10	36
YGS, China	24	36
YHT, China	19	36
YKM, China	40	36
YLO, China	42	36
YLT, China	26	36
YMB, China	6	36
YMI, China	32	36
YPA, China	32	36
YTU, China	41	36
YWU, China	31	36
YXB, China	11	36
YYM, China	27	36
Q_Tib, China	76	9
G_Tib, China	83	9
XZ-Lhoba, China	50	Our unpublished data
XZ-Monba, China	31	Our unpublished data
wanyan, China	46	Our unpublished data
Japanese, Japan	50	37
Japanese, Japan	162	38
Japanese, Japan	211	39
Gifu, Japan	137	40
Japanese, Japan	150	34
Japanese, Japan	100	41
Japanese, Japan	124	42
Japanese, Japan	62	43
Ainu, Japan	50	43
Pyukyuan, Japan	50	43
Achang-YN, China	60	Our unpublished data

Bai-YN, China	21	Our unpublished data
Bai-YN, China	19	Our unpublished data
Blang-YN, China	90	Our unpublished data
Dai-YN, China	46	Our unpublished data
Dai-YN, China	24	Our unpublished data
Dai-YN, China	30	Our unpublished data
Dai-YN, China	27	Our unpublished data
Deang-YN, China	52	Our unpublished data
Drung -YN, China	35	Our unpublished data
Hani-YN, China	9	Our unpublished data
Hani-YN, China	39	Our unpublished data
Hani-YN, China	18	Our unpublished data
Jingpo-YN, China	60	Our unpublished data
Jino-YN, China	51	Our unpublished data
Lahu-YN, China	26	Our unpublished data
Lahu-YN, China	36	Our unpublished data
Lisu-YN, China	31	Our unpublished data
Lisu-YN, China	20	Our unpublished data
Lisu-YN, China	18	Our unpublished data
Naxi-YN, China	62	Our unpublished data
Naxi-YN, China	6	Our unpublished data
Nu-YN, China	40	Our unpublished data
Pumi-YN, China	19	Our unpublished data
Pumi-YN, China	25	Our unpublished data
Tibetan-YN, China	11	Our unpublished data
Tibetan-YN, China	54	Our unpublished data
Wa-YN, China	14	Our unpublished data
Yao-YN, China	8	Our unpublished data
Yi-YN, China	28	Our unpublished data
Yi-YN, China	67	Our unpublished data
Blang-YN, China	48	Our unpublished data
DL-YN, China	48	Our unpublished data
Dongxiang-GS, China	63	Our unpublished data
GL-GZ, China	102	Our unpublished data
GX-Dong, China	100	Our unpublished data
GX-Maonan, China	48	Our unpublished data
GX-Mulam, China	48	Our unpublished data
GX-Yao, China	29	Our unpublished data
GZ-Bouyei, China	85	Our unpublished data
GZ-Miao, China	35	Our unpublished data
GZ-She, China	49	Our unpublished data
GZ-Shui, China	64	Our unpublished data
GZ-Yao, China	40	Our unpublished data
Han-GS, China	43	Our unpublished data

Han-JS, China	1	Our unpublished data
Han-NX, China	111	Our unpublished data
Han-SX, China	2	Our unpublished data
Hainan-Li, China	59	Our unpublished data
HN-Miao, China	49	Our unpublished data
HN-Tujia, China	46	Our unpublished data
HN-Yao, China	52	Our unpublished data
Hui-QH, China	33	Our unpublished data
Hui-GS, China	21	Our unpublished data
IM-Buryat, China	59	Our unpublished data
IM-Man, China	122	Our unpublished data
IM-Xibe, China	49	Our unpublished data
Li-HI, China	216	Our unpublished data
LN-Man, China	31	Our unpublished data
Han-BJ, China	45	Our unpublished data
Han-QH, China	142	Our unpublished data
QH-Tu, China	64	Our unpublished data
QJ-YN, China	40	Our unpublished data
Sala-QH, China	16	Our unpublished data
XM, China	40	Our unpublished data
YN-Bai, China	66	Our unpublished data
YN-Blang, China	37	Our unpublished data
YN-Dai, China	85	Our unpublished data
YN-Hani, China	44	Our unpublished data
YN-Jino, China	31	Our unpublished data
YN-Kucong, China	34	Our unpublished data
YN-Lisu, China	43	Our unpublished data
YN-Mosuo, China	64	Our unpublished data
YN-Naxi, China	56	Our unpublished data
YN-Pumi, China	59	Our unpublished data
YN-Yi, China	90	Our unpublished data
Zhuang-GX, China	79	Our unpublished data
Han-GD, China	105	44
HHT-NM, China	107	45
LQ-NM, China	48	45
TH-YN, China	46	45
AC, China	6	46
BG, China	32	46
BU, China	31	46
CL, China	30	46
CU, China	30	46
CX, China	25	46
CY, China	12	46
DA, China	56	46

DG, China	40	46
DN, China	10	46
GA, China	42	46
HL, China	34	46
HS, China	30	46
JM, China	27	46
LC, China	30	46
LG, China	31	46
LL, China	4	46
LQ, China	25	46
MK, China	33	46
ML, China	39	46
MN, China	32	46
MO, China	29	46
MQ, China	17	46
PO, China	34	46
PY, China	30	46
RG, China	31	46
TN, China	30	46
WG, China	14	46
WS, China	33	46
YR, China	15	46
HAN- Chaoshan, GD, China	102	47
HAN- Meizhou, GD, China	170	47
Northern-Han, China	60	48
Japanese_Honshu, Japan	82	48
Indonesian, Indonesian	54	48
Koryak-KMAN, Koryak	110	48
Japanese_Kyushu, Japan	104	48
Malaysian-MAL, Malaysian	52	48
Nivkhi, Nivkhi	57	48
Okinawa, Japan	45	48
Philippine, Philippine	29	48
Philippine, Philippine	30	48
Kam, China	72	49
Laka, China	67	49
Mien, China	29	49
Mulam, China	27	49
Pinghua_Han, China	39	49
Pinghua_Han, China	48	49
Pinghua_Han, China	111	49
Zhuang_GB, China	54	49
Zhuang_GB, China	4	49
Zhuang_GB, China	9	49

Zhuang_GB, China	10	49
Bunun, China	18	50
Atayal, China	18	50
Amis, China	21	50
Paiwan, China	21	50
Undefined, Taiwan China	64	51
Han-JS, China	99	Our unpublished data
HSK-XJ, China	44	Our unpublished data
Kirgiz-XJ, China	60	Our unpublished data
Mg-XJ, China	96	Our unpublished data
Tajik-XJ, China	56	Our unpublished data
Uyu-XJ, China	274	Our unpublished data
Uzbek-XJ, China	46	Our unpublished data
Han-HLJ, China	180	Our unpublished data
Han-SX, China	220	Our unpublished data
Han-JL, China	199	Our unpublished data
Han-LN, China	191	Our unpublished data
Han-HN, China	1502	52
Han-YN, China	396	Our unpublished data
Han-SN, China	398	Our unpublished data
Han-AH, China	375	Our unpublished data
Han cancer of the esophagus, China	190	53
Han cancer of the esophagus, China	106	Our unpublished data
Han-SC, China	556	Our unpublished data
Han-SC, China	214	Our unpublished data
Han-SC, China	312	Our unpublished data
Koreans 103, South Korea	103	54
ZA_Chamdo_Ji, China	29	55
ZA_Lhasa_Ji, China	44	55
ZA_Nyingchi_Ji, China	52	55
ZA_Shigatse_Ji, China	29	55
ZA_Shannan_Ji, China	55	55
ZA_Nakchu_Ji, China	5	55
Tib_Ji, China	73	55
Tibetan, China	156	56
Korean, South Korea	694	57
HA, Shandong, China	253	Our unpublished data
HA, Neimeng, China	141	Our unpublished data
Dongbei, China	249	Our unpublished data
QI, Sichuan, China	60	Our unpublished data
Southeast Asia		
Vietnamese, Vietnam	42	5
Thais, Thailand	40	5
Vietnamese, Vietnam	187	58

Burman, Karen, Myanmar	327	59
Thai LHON, Thailand	42	Tharaphan et al. GenBank DQ149024-DQ149065
Indonesian, Sumatra, Pekanbaru, Indonesia	55	60
Indonesian, Sumatra, Medan, Indonesia	44	60
Malaysian, Semang, Malaysia	263	60
Malaysian, Melayu, Malaysia	4	60
Vietnamese, Vietnam	2	8
Samoan, Samoa	9	8
Philippines, Philippines	3	8
Borneo, Borneo	13	8
Java, Java	2	8
Burmans, Myanmar	32	Our unpublished data
Burmans, Myanmar	54	Our unpublished data
Burmans, Myanmar	51	Our unpublished data
Burmans, Myanmar	122	Our unpublished data
Burmans, Myanmar	69	Our unpublished data
Burmans, Myanmar	72	Our unpublished data
Chin, Myanmar	58	Our unpublished data
Chin, Myanmar	13	Our unpublished data
Chin, Myanmar	187	Our unpublished data
Naga, Myanmar	30	Our unpublished data
Naga, Myanmar	39	Our unpublished data
Naga, Myanmar	32	Our unpublished data
Rakhine, Myanmar	24	Our unpublished data
Rakhine, Myanmar	63	Our unpublished data
Akha, Thailand	91	61
Lahu, Thailand	39	61
Lisu, Thailand	25	61
Lisu, Thailand	42	61
Lisu, Thailand	53	61
Mussur, Thailand	21	61
Cham, Vietnam	168	62
Kinh, Vietnam	139	62
Thai, Thailand	190	63
Thai, Thailand	32	11
Cambodia, Cambodia	31	64
Thai, Thailand	30	65
Thai, Thailand	44	65
Lao Song, Thailand	25	65
ChB, Thailand	20	66
Khm, Thailand	22	66

Th_K, Thailand	32	66
Phuthai, Thailand	25	65
Chong, Thailand	25	65
Bangka, Sumatra, Indonesia	34	67
Batek, Malaysia	45	67
Jahai, Malaysia	39	67
Medan, Sumatra, Indonesia	42	67
Melayu, Malaysia	6	67
Mendriq, Malaysia	31	67
Padang, Malaysia	24	67
Palembang, Sumatra, Indonesia	50	67
Pekanbaru, Sumatra, Indonesia	52	67
Semai, Malaysia	6	67
Semelai, Malaysia	61	67
Temiar, Malaysia	46	67
Temuan, Malaysia	33	67
Philippine, Philippines	61	50
Java, Indonesia	46	50
Banjarmasin, Indonesia	89	50
Kota Kinabalu, Malaysia	61	50
Manado, Indonesia	89	50
Palu, Indonesia	38	50
Ujung Padang, Indonesia	46	50
Toraja, Indonesia	64	50
Bali, Indonesia	82	50
Mataram, Indonesia	44	50
Flores, Indonesia	2	50
Waingapu, Indonesia	50	50
Alor, Malaysia	45	50
Ambon, Indonesia	43	50
Luzon, Philippines	168	51
Visayas, Philippines	103	51
Mindanao, Philippines	70	51
Mal, Malaysia	124	68
HA, Singapore	205	69
Sakai, Thailand	20	65
Mussur, Thailand	21	65
White Karen, Thailand	40	61
Red Karen, Thailand	39	61
BK, Thailand	13	66
NUL, Thailand	17	66
Benu, Malaysia	10	70
Gopeng, Malaysia	22	70
Kuala Kurau, Malaysia	17	70

Lembah Bujang, Malaysia	26	70
Lenggeng, Malaysia	12	70
Machang, Malaysia	24	70
Kota Bharu, Malaysia	5	70
Muar, Malaysia	17	70
Parit Buntar, Malaysia	16	70
Parit Buntar, Malaysia	1	70
Pontian, Malaysia	20	70
Rantau Panjang, Malaysia	32	70
Semerah, Malaysia	13	70
Sri Menanti, Malaysia	22	70
Yan, Malaysia	11	70
Laos, Laos	214	71
South Asia		
Tipperah, India	20	72
Indians, Jammu and Kashmir, India	7	Bhat et al. GenBank AN. AY642034-AY642040
Indian, India	12	Darvishi et al. GenBank DQ143184-DQ143195
Indian, north India	4	Darvishi et al. GenBank DQ176757-DQ176760
Chakma, India	92	Our unpublished data
Ralt, India	104	Our unpublished data
Kuki, India	45	Our unpublished data
Adi, India	45	73
Apatani, India	26	73
Apatani, India	21	73
Naga, India	43	73
Nishi, India	44	73
Garo, India	76	74
Lyngnga, India	74	74
Nongtrai, India	27	74
Maram, India	60	74
Bhoi, India	29	74
Khynriam, India	82	74
War_Khas, India	29	74
Pnar, India	51	74
War_Jaint, India	17	74
Pakistani, Pakistan	100	75
Baluch, Pakistan	39	75
Brahui, Pakistan	38	75
Gujarati, India	34	75
Hazara, Pakistan	23	75
Hunza Burusho, Pakistan	44	75

Kalash, Pakistan	44	75
Makrani, Pakistan	33	75
Mazandarian, Pakistan	44	75
Pathan, Pakistan	44	75
Sindhi, Pakistan	23	75
Moor, Sri Lanka	43	76
Sinhalese, Sri Lanka	59	76
Indo-European, India	94	77
Dravidian, India	17	77
Kadar, India	7	78
Mixed MD, MDU, TH, India	96	Our unpublished data
Mixed DG, AMK, ARA, CNP, ERI, KAM, India	400	Our unpublished data
Mixed KAN, KAS, NAT, TNP, ODC, India	263	Our unpublished data
Mixed NGL, VRU,VEM, VKD, TN, MX, India	190	Our unpublished data
Baduga, India	2	79
Irula, India	16	72, 7[illegible]
Kota, India	25	73
Kurumba, India	8	73
Kurumba Betta, India	19	73
Kurumba Mullu, Mulla Krumba, India	15	73
Oorali, India	3	79
Sakkili, India	6	79
Soligas, India	12	73
Malayalam , India	3	80
Cochin, India	52	76
Cochin Jews, India	37	76
Kattunaiken, India	16	73
Kuruchian, India	46	73
Kuruman Mullu, Mullu Kurunan, India	37	73
Paniya, India	11	73
Mixed TVM, India	64	Our unpublished data
Havik, India	38	78
Mukri, India	42	78
Kannada, India	5	80
Koragas, India	31	73
Kuruva, India	25	81
Kurumba Jenu, India	6	73
Yerava, India	53	73
Bhovi, India	30	82
Gowda, India	37	82
Brahmin, India	29	82

Lingayat, India	17	82
Christians, India	21	82
Muslim, India	24	82
Siddi, India	7	83
Telugu, Andhra Pradesh, Ind, TE, India	15	80, 8[illegible], 85
Lambadi, India	75	84
Andh, AndhSA, AndhX, India	95	73, 8[illegible]
Brahmin, India	34	84
Kapu, KTK, KP, India	67	84, 8[illegible]
Madiga, India	24	84
Yadava, India	40	84
Wadabahija, India	8	84
Mala, India	23	84
Kshatriya, India	9	84
Relli, India	20	84
Jalari, India	7	84
Vysya, India	9	84
Chenchu, India	96	88
Koya, India	81	88
Erukula, India	5	79
Yanadi, India	10	79
Thogataveera, India	84	Our unpublished data
Reddy, India	68	Our unpublished data
Pardhi, India	38	73
Thoti, India	39	73
Akhutota, India	22	87
PantaWatkins, India	29	87
Pokanati, India	36	87
Vanne, India	26	87
Irula, India	19	89
Chenchu, India	75	90
Kolam, India	128	90
Gond, India	75	90
Naikpod, N, NP, Z, India	83	86
Pardhan, D, I, J, S, W, India	170	86
Chitpavan Brahmin, India	17	91
Desasth Brahmin, India	15	91
Brahmin, India	9	92
Konkanastha Brahmin, India	50	76
Dhangar, India	16	91
Maratha, India	30	91-93
Maharashtra, Marathi, India	29	80, 8[illegible]
Nav-Baudh, India	17	92, 9[illegible]
Parsi, India	37	76

Bohra, India	5	93
Irani, India	5	93
Korku, India	9	93
Madia Gond, India	13	93
Kolam, India	8	93
Kathodi, India	1	79
Koli, India	6	79
Sikh, India	23	73
Gujarat, India	61	75, 76
Brahmin, India	29	94
Khatri, India	11	94
Jat Singh, India	27	76, 94
Scheduled caste, India	19	76, 94
Lobana, India	49	84
Brahmin, India	18	76
Kshatriya, India	22	76
PunjabP, PBAN, PHC, PNIR, PP, PPUR, PPUS, PRAJ, PRAM, PSUR, PSUS, PUSH, India	42	77, 85
Bhoksa, India	22	84
Tharu, India	31	76, 84
MundaMun, India	4	79
KoriKor, India	1	79
Rohidas, India	1	79
Yadava, India	6	79
Bhargava, India	165	Our unpublished data
Chaturvedi, India	82	Our unpublished data
Brahmin Mixed, India	146	Our unpublished data
Uttar Pradesh Brahmin, India	23	76
Rajput, India	14	79
Lodhe, India	1	79
HI, U, Uttar Pradesh, USIN, UKIR, UANI, UBHA, UBOB, UCHA, UD, UHC, UJEA, India	93	77, 80, 84
Delhi, India	10	85
Kanet, India	29	76
Rajput, Rajsthan, India	33	76, 84
Kashmir, India	13	84
Baluch, Pakistan	14	75
Brahui, Pakistan	12	75
Hazara, Pakistan	12	75
Hunza Burusho, Pakistan	23	75
KarachiKAR, Pakistan, UR, Pakistan	74	75, 80, 84
Parsi, Pakistan	23	75

Pathan, Pakistan	23	75
SindhiSI, Pakistan	14	75
Pushtoons, Pakistan	16	73
Makrani, Pakistan	8	73
BI, BD, BHC, BSUY, BVIJ, Bihar, India	43	77, 80, 84
BhumijBhuJ, BM, India	60	94, 95
Ho, India	20	95
Kharia, India	39	87, 95
KanwarKan, India	6	79
SatnamiSat, India	3	79
SanthalSa, India	45	79, 87
AsurAS, India	30	87
Munda, India	23	87
BirhorBir, India	189	Our unpublished data
Munda, India	169	Our unpublished data
Oran, India	169	Our unpublished data
Phariya, India	160	Our unpublished data
Bharia, India	14	79
Gond, Madia Gond, India	4	79, 89
Oran, India	16	79
Muria, India	12	72
Brahmin, India	16	96
Gope, India	17	96
Juang, India	20	96
Karan, India	14	96
Khandayat, India	14	96
Paroja, India	21	96
Saora, India	17	96
Orisa, India	2	84
BE, Ben, Bengal, India	15	79, 80, 84
Bengal Brahmin, India	36	76
Bengal Sudra, India	8	76
Rajbhansi, SW, Ra, RJ, India	249	Our unpublished data
Kurmi, India	55	76
Lodha, Lodhar, India	68	72, 76
Munda, India	6	72
Santhal, India	14	72
Dhimal, India	75	Our unpublished data
Kol, India	73	Our unpublished data
Lachung, India	35	Our unpublished data
Mech, India	45	Our unpublished data
Munda, India	40	Our unpublished data
Oran, India	98	Our unpublished data

Phariya, India	175	Our unpublished data
Rabha, India	49	Our unpublished data
Santhal, India	83	Our unpublished data
Toto, India	11	Our unpublished data
Mixed, MX, WBX, India	409	Our unpublished data
Bodo, India	65	Our unpublished data
Muslim, India	71	Our unpublished data
Bangladesh, Bangladesh	1	84
Bang, Bangladesh	27	73
Mixed, Bangladesh	143	Our unpublished data
Great Andamanese, India	20	97
Jarawa, India	4	97
Onge, India	62	97
Nicobarese, India	46	97, 98
Ao, India	19	79
Mixed, Bhutan	47	Our unpublished data
Mixed, Nepal	61	Our unpublished data
Ladakhis-Bud, Kashmir	23	99
Ladakhis-Mus, Kashmir	9	99
Shia Muslim, India	120	100
Sunni Muslim, India	131	100
Dawoodi Bohra, India	62	100
Dawoodi Bohra, India	50	100
Mappla, India	61	100
Iranian Shia, India	48	100
Tharu-C I, Nepal	57	101
Tharu-C II, Nepal	76	101
Tharu-E, Nepal	40	101
Nepal-Kathamandu, Nepal	200	Our unpublished data
Nepal-East Nepal, Nepal	46	Our unpublished data
Tamang, Nepal	46	56
Newar, Nepal	67	56
Kathmandu, Nepal	77	56
North Asia		
Besermyans, Udmurtia	41	Grosheva et al. GenBank AN. KF380839-KF380879
Mongolian, Mongolia	47	5
Volga-Turkic, Russia	2	Zaporozhchenko et al. GenBank AN. DQ656492-DQ656493
Aznakaevo, Aznakaevo	71	102
Buinsk, Buinsk	125	102
Mongoloid, Mongolia	103	103
AEG, Mongolia	39	104
PDEG, Mongolia	124	104

PDM, Mongolia	104	104
PDY, Mongolia	44	104
Mongoloid, Mongolian	46	104
Buryat, South Siberia	40	105
Tuvinian, South Siberia	36	105
Russian, north-eastern Siberia	50	106
Ukrainian, north-eastern Siberia	18	106
Altaian, South Siberia	110	107
Khakassian, South Siberia	53	107
Buryat, South Siberia	91	107
Sojot, South Siberia	30	107
Todjin, South Siberia	48	107
Tuvinian, South Siberia	90	107
Tofalar, South Siberia	58	107
Buryat, Ulan-Ude	126	108
Yakut, Yakutsk	117	108
Yakut, northeastern Siberia	191	109
Even, Russia	65	110
Koryak, Russia	35	110
Yakutian, Russia	22	110
Kets, Sulomai and Turukhansk	38	111
Ngana-sans, Dudinka	24	111
Ba, bashkir	221	112
Ch, chuvash	55	112
Er, erza-moksha	102	112
Ko, komi	138	112
Ma, mari	139	112
Ta, tatar	228	112
Ud, udmurt	102	112
TB, Southern Siberia	72	113
TV, Southern Siberia	96	113
BR, Southern Siberia	25	113
TF, Southern Siberia	46	113
EV, Southern Siberia	37	113
NG, Southern Siberia	33	113
UL, Southern Siberia	87	113
NV, Southern Siberia	56	113
UD, Southern Siberia	46	113
Mansi, Northwest Siberia	98	111
Nga, Arctic Siberians	39	114
Ind, Arctic Siberians	82	114
Kolyma, Arctic Siberians	18	114
Chv, Arctic Siberians	32	114
Chu, Arctic Siberians	182	114

Sir, Arctic Siberians	37	114
Cha, Arctic Siberians	50	114
Nau, Arctic Siberians	39	114
Ale, Arctic Siberians	36	114
Buryat, Russia	61	48
Even, Russia	35	48
Chelkan, Northern Altaians	91	115
Kumandin, Northern Altaians	52	115
Tubalar, Northern Altaians	71	115
Altai-kizhi , Southern Altaians	276	115
Barghuts, Hulun Buir Aimak ,Inner Mongolia, China	149	116
Altaian Kazakhs, Kosh-Agach district of Altai Republic	98	116
Mongolians, Ulaanbaatar, Mongolia	47	54
Kalmyks, Kalmyk Republic	110	54
Buryats, Buryat Republic	295	54
Khamnigans, Buryat Republic	99	54
Tuvinians, Tuva Republic	105	54
East Evenks, Buryat Republic	45	54
West Evenks, Krasnoyarsk	73	54
Yakuts, Sakha	36	54
Shors, Kemerovo	82	54
Khakassians, Khakassian Republic	57	54
Altaians-Kizhi, South Altai	90	54
Telenghits, South Altai	71	54
Teleuts, Kemerovo	53	54
Chukchi, Anadyr, Chukotka Autonomous Okrug	15	54
Khanty, Lower Ob-river valley	106	117
Mansi, Lower Ob-river valley	63	117
Complete sequences of C and D, North Asia	182	118
Complete sequences of North Asia rare haplogroups	55	116
Tubalar, Altai region	144	119
Evens, northeastern Siberia	87	119
Ulchi, Russian Far East	160	119
Central Asia		
Bukharan Arabs, Central Asia	20	120
Crimean Tatars, Central Asia	20	120
Dungans, Central Asia	16	120
Iranians, Central Asia	20	120
Karakalpaks, Central Asia	20	120

Kazaks, Central Asia	20	120
Khoremian Uzbeks, Central Asia	20	120
Kyrgyz, Central Asia	20	120
Tajiks, Central Asia	20	120
Turkmen, Central Asia	20	120
Uighurs, Central Asia	16	120
Uzbeks, Central Asia	20	120
Kazakh, Kazakhstan	55	120
Kirghiz, Kirghizstan	47	120
Kirghiz, Kirghizstan	48	120
Uighur, Kazakhstan	55	120
Kurdish, Turkmenistan	32	75
Shugnan, Tajikistan	44	75
Turkmen, Turkmenistan	41	75
Uzbek, Uzbekistan	42	75
FER, Uzbekistan	53	121
KAR, Uzbekistan	46	121
KAZ, Kazakhstan	256	121
KYR, Kyrgyzstan	249	121
QAS, Uzbekistan	75	121
RUS, Russia	151	121
TAJ, Tajikistan	244	121
TAS, Uzbekistan	55	121
TUR, Turkmenistan	249	121
XOR, Uzbekistan	99	121
KAR, Uzbekistan	55	122
KAZ, Kazakhstan	50	122
OTU, Uzbekistan/Turkmenistan border	53	122
TUR, Uzbekistan/Turkmenistan border	51	122
UZB, Karakalpakia	40	122
Kazakhstan, Kazakhstan	27	123
Tajiks, Tajikistan	44	54
West Asia		
Kurdish Muslims, Baghdad, Iraq	15	124
Assyrian Christians, Baghdad, Iraq	22	124
Arab Muslims, Baghdad, Iraq	128	124
Mandaean Arabs, Baghdad, Iraq	17	124
Kuwaiti, Kuwait	381	125
Yemeni, Yemen	50	1
Bedouin, Near East	58	3
Cherkes, Near East	8	3
Druze, Near East	77	3
Palestinian, Near East	110	3
Turkish, Turkey	50	75

BAL, Georgia	20	75
CHE, Georgia	18	75
GEO, Georgia	20	75
Gilaki, Iran	37	75
Kurdish, Iran	20	75
Lur, Iran	17	75
Mazandarian, Iran	21	75
Persian, Iran	42	75
Turkish, Azerbaijan	40	75
AFG, Afghanistan	98	121
Persians, eastern Iran	82	54
Kurds, northwestern Iran	25	54
Europe		
Basque, Navarre, Spain	110	126
Autochthonous Basque lineages, Spain	56	Cardoso et al. GenBank AN. JX669072-JX669127
Autochthonous Basque lineages, Spain	51	Cardoso et al. GenBank AN. JX669021-JX669071
Basque, Northern Navarre, Spain	100	127
Basque, Basque Country northern Spain	106	127
Basque, Franco-Cantabrian Region	35	127
Basque, Basque Country northern Spain	55	127
Patients of sporadic prostate cancer, Southwest Europe	239	128
Controls of sporadic prostate cancer, Southwest Europe	150	128
Patients with schizophrenia and bipolar Disorder, Italy	89	129
T2DM patients, Marche, Italy	466	130
Controls, Marche, Italy	438	130
French , France	142	131
Veneto speakers, Barco, Veneto, Italy	30	132
Veneto speakers, Barco, Veneto, Italy	38	132
Spanish, Pas Valley, Spain	61	133
Etruscans, Tuscany, Italy	322	134
Greek, northern Greece	319	135
Cyprus, Cyprus	91	135
Remains from the Alps, Italy	3	136
Italian, Trexenta, Sardinia, Italy	47	Calo et al. GenBank 81669-DQ0DQ081715
Italian, Tuscany, Italy	61	Varesi et al. GenBank 81608-DQ0DQ081668

Italian, San Pietro, Sardinia, Italy	44	Varesi et al. GenBank DQ081564-DQ081607
Italian, Sant Antioco, Sardinia, Italy	42	Falchi et al. GenBank DQ081522-DQ081563
Italian, Gallura, Sardinia, Italy	50	Varesi et al. GenBank DQ081420-DQ081469
French, South Corsica, France	53	Giovannoni et al. GenBank DQ081367-DQ081419
Spanish, Baleares, Spain	67	Varesi et al. GenBank DQ081300-DQ081366
Andalusian, Spain	66	Via et al. GenBank DQ081234-DQ081299
Italian, Nuoro, Sardinia, Italy	51	Varesi et al. GenBank DQ067827-DQ067877
Ancient Cumanians, Hungary	11	137
Magyars/Hungarians, Carpathian basin	35	Kalmar et al. GenBank AF487581-AF487615
Nefedjevo inhabitant, Russia	8	Buzhilova et al. GenBank AF466686-AF466693
Belorussian, Belarus	55	Belyaeva et al. GenBank AY005336-AY005390
Scotch, Scotland, UK	1341	138
Norse, Norway	323	138
Pol, Northern Poland	436	139
Rus, Russia	201	139

Supplementary References

1 Non, A. L., Al-Meeri, A., Raaum, R. L., Sanchez, L. F. & Mulligan, C. J. Mitochondrial DNA reveals distinct evolutionary histories for Jewish populations in Yemen and Ethiopia. *Am. J. Phys. Anthropol.* **144**, 1-10 (2011).

2 Behar, D. M. *et al.* The matrilineal ancestry of Ashkenazi Jewry: portrait of a recent founder event. *Am. J. Hum. Genet.* **78**, 487-497 (2006).

3 Behar, D. M. *et al.* Counting the founders: the matrilineal genetic ancestry of the Jewish Diaspora. *PLoS ONE* **3**, e2062 (2008).

4 Brandstätter, A. *et al.* Mitochondrial DNA control region variation in Ashkenazi Jews from Hungary. *Forensic Sci Int Genet* **2**, e4-e6 (2008).

5 Jin, H.-J., Tyler-Smith, C. & Kim, W. The peopling of Korea revealed by analyses of mitochondrial DNA and Y-chromosomal markers. *PLoS ONE* **4**, e4210 (2009).

6 Irwin, J. A. *et al.* Investigation of heteroplasmy in the human mitochondrial DNA control region: a synthesis of observations from more than 5000 global population samples. *J. Mol. Evol.* **68**, 516-527 (2009).

7 Xu, Z. *et al.* Mitochondrial DNA evidence for a diversified origin of workers building mausoleum for first emperor of China. . *PLoS ONE* **3**, e3275 (2008).

8 Koji Lum, J. & Cann, R. L. mtDNA lineage analyses: origins and migrations of Micronesians

and Polynesians. *Am. J. Phys. Anthropol.* **113**, 151-168 (2000).

9 Zhao, M. *et al.* Mitochondrial genome evidence reveals successful Late Paleolithic settlement on the Tibetan Plateau. *Proc. Natl. Acad. Sci.* **106**, 21230-21235 (2009).

10 Qin, Z. *et al.* A mitochondrial revelation of early human migrations to the Tibetan Plateau before and after the last glacial maximum. *Am. J. Phys. Anthropol.* **143**, 555-569 (2010).

11 Yao, Y. G. *et al.* Genetic relationship of Chinese ethnic populations revealed by mtDNA sequence diversity. *Am. J. Phys. Anthropol.* **118**, 63-76 (2002).

12 Wen, B. *et al.* Analyses of genetic structure of Tibeto-Burman populations reveals sex-biased admixture in southern Tibeto-Burmans. *Am. J. Hum. Genet.* **74**, 856-865 (2004).

13 Yao, Y. G. & Zhang, Y. P. Phylogeographic analysis of mtDNA variation in four ethnic populations from Yunnan Province: new data and a reappraisal. *J. Hum. Genet.* **47**, 311-318 (2002).

14 Qian, Y. P. *et al.* Mitochondrial DNA polymorphisms in Yunnan nationalities in China. *J. Hum. Genet.* **46**, 211-220 (2001).

15 Yao, Y. G., Kong, Q. P., Wang, C. Y., Zhu, C. L. & Zhang, Y. P. Different matrilineal contributions to genetic structure of ethnic groups in the Silk Road region in China. *Mol. Biol. Evol.* **21**, 2265-2280 (2004).

16 Liu, X. & Li, S. Polymorphism of mitochondrial DNA D-loop region in Chinese Baoan ethnic group. *Journal of the Fourth Military Medical University* **20**, 004 (2003).

17 Liu, X., Chen, T. & Li, S. Sequence polymorphism of human mitochondrial DNA control region in Chinese Dongxiang unrelated individuals. *Journal of Medical Colleges of PLA* **19** (2004).

18 Liu, X. & Li, S. Study on polymorphisms of mitochondrial DNA D-loop region in the Sala population in China. *J Xi'an Jiaotong Univ* **25** (2004).

19 Wang, W., Wise, C., Baric, T., Black, M. L. & Bittles, A. H. The origins and genetic structure of three co-resident Chinese Muslim populations: the Salar, Bo'an and Dongxiang. *Hum. Genet.* **113**, 244-252 (2003).

20 Liu, X. & Li, S. Mitochondrial DNA Polymorphism in control region from Chinese Yugu population. *J Xi'an Jiaotong Univ* **16** (2004).

21 Li, C. *et al.* Evidence that a West-East admixed population lived in the Tarim Basin as early as the early Bronze Age. *BMC Biol.* **8**, 15 (2010).

22 Xie, C. Z. *et al.* Quantification Polymerase Chain Reaction Designs to Analyze the Ancient Deoxyribonucleic Acid of Xiaohe Cemetery, Xinjiang. *Chinese Journal of Analytical Chemistry* **35**, 5 (2007).

23 Cui, Y., Li, C., Gao, S., Xie, C. & Zhou, H. Early Eurasian migration traces in the Tarim Basin revealed by mtDNA polymorphisms. *Am. J. Phys. Anthropol.* **142**, 558-564, (2010).

24 Cui, Y. *et al.* 新疆塔里木盆地早期铁器时代人群的母系遗传结构分析. *Chinese Science Bulletin* **54**, 2912-2919 (2009).

25 He, H. *et al.* Study on mtDNA polymorphism of ancient human bone from Hami of Xinjiang, Chian 3200BP. *Acta Anthropologica Sinica* **22** (2003).

26 Kong, Q. P. *et al.* Mitochondrial DNA sequence polymorphisms of five ethnic populations from northern China. *Hum. Genet.* **113**, 391-405 (2003).

27 Zhang, Y. J., Xu, Q. S., Zheng, Z. J., Lin, H. Y. & Lee, J. B. Haplotype diversity in mitochondrial DNA hypervariable region I, II and III in northeast China Han. *Forensic Sci. Int.*

149, 267-269 (2005).
28 Wen, B. *et al.* Genetic evidence supports demic diffusion of Han culture. *Nature* **431**, 302-305 (2004).
29 Yao, Y. G., Kong, Q. P., Bandelt, H.-J., Kivisild, T. & Zhang, Y. P. Phylogeographic differentiation of mitochondrial DNA in Han Chinese. *Am. J. Hum. Genet.* **70**, 635-651 (2002).
30 Yao, Y. G., Kong, Q. P., Man, X. Y., Bandelt, H.-J. & Zhang, Y. P. Reconstructing the evolutionary history of China: a caveat about inferences drawn from ancient DNA. *Mol. Biol. Evol.* **20**, 214-219 (2003).
31 Tajima, A. *et al.* Mitochondrial DNA polymorphisms in nine aboriginal groups of Taiwan: implications for the population history of aboriginal Taiwanese. *Hum. Genet.* **113**, 24-33 (2003).
32 Tsai, L. *et al.* Sequence polymorphism of mitochondrial D-loop DNA in the Taiwanese Han population. *Forensic Sci. Int.* **119**, 239-247 (2001).
33 Kivisild, T. *et al.* The emerging limbs and twigs of the East Asian mtDNA tree. *Mol. Biol. Evol.* **19**, 1737-1751 (2002).
34 Nishimaki, Y. *et al.* Sequence polymorphism in the mtDNA HV1 region in Japanese and Chinese. *Leg Med* **1**, 238-249 (1999).
35 Oota, H. *et al.* Extreme mtDNA homogeneity in continental Asian populations. *Am. J. Phys. Anthropol.* **118**, 146-153 (2002).
36 Wen, B. *et al.* Genetic structure of Hmong-Mien speaking populations in East Asia as revealed by mtDNA lineages. *Mol. Biol. Evol.* **22**, 725-734 (2005).
37 Koyama, H. *et al.* Mitochondrial sequence haplotype in the Japanese population. *Forensic Sci. Int.* **125**, 93-96 (2002).
38 Imaizumi, K., Parsons, T. J., Yoshino, M. & Holland, M. A new database of mitochondrial DNA hypervariable regions I and II sequences from 162 Japanese individuals. *Int. J. Legal Med.* **116**, 68-73 (2002).
39 Maruyama, S., Minaguchi, K. & Saitou, N. Sequence polymorphisms of the mitochondrial DNA control region and phylogenetic analysis of mtDNA lineages in the Japanese population. *Int. J. Legal Med.* **117**, 218-225 (2003).
40 Nagai, A., Nakamura, I., Shiraki, F., Bunai, Y. & Ohya, I. Sequence polymorphism of mitochondrial DNA in Japanese individuals from Gifu Prefecture. *Leg Med* **5**, S210-S213 (2003).
41 Seo, Y., Stradmann-Bellinghausen, B., Rittner, C., Takahama, K. & Schneider, P. M. Sequence polymorphism of mitochondrial DNA control region in Japanese. *Forensic Sci. Int.* **97**, 155-164 (1998).
42 Mabuchi, T., Susukida, R., Kido, A. & Oya, M. Typing the 1.1 kb control region of human mitochondrial DNA in Japanese individuals. *J. Forensic Sci.* **52**, 355-363 (2007).
43 Horai, S. *et al.* mtDNA polymorphism in East Asian Populations, with special reference to the peopling of Japan. *Am. J. Hum. Genet.* **59**, 579 (1996).
44 Chen, F. *et al.* Analysis of mitochondrial DNA polymorphisms in Guangdong Han Chinese. *Forensic Sci Int Genet* **2**, 150-153 (2008).
45 Cheng, B. *et al.* Genetic imprint of the Mongol: signal from phylogeographic analysis of mitochondrial DNA. *J. Hum. Genet.* **53**, 905-913 (2008).

46 Li, H. *et al.* Mitochondrial DNA diversity and population differentiation in southern East Asia.*Am. J. Phys. Anthropol.* **134**, 481-488 (2007).

47 Wang, W. Z. *et al.* Tracing the origins of Hakka and Chaoshanese by mitochondrial DNA analysis. *Am. J. Phys. Anthropol.* **141**, 124-130 (2010).

48 Tajima, A. *et al.* Genetic origins of the Ainu inferred from combined DNA analyses of maternal and paternal lineages. *J. Hum. Genet.* **49**, 187-193 (2004).

49 Gan, R. J. *et al.* Pinghua population as an exception of Han Chinese's coherent genetic structure.*J. Hum. Genet.***53**, 303-313 (2008).

50 Hill, C. *et al.*A mitochondrial stratigraphy for island southeast Asia.*Am. J. Hum. Genet.***80**, 29-43 (2007).

51 Tabbada, K. A. *et al.* Philippine mitochondrial DNA diversity: a populated viaduct between Taiwan and Indonesia? *Mol. Biol. Evol.* **27**, 21-31 (2010).

52 Zhang, W. *et al.*A Matrilineal Genetic Legacy from the Last Glacial Maximum Confers Susceptibility to Schizophrenia in Han Chinese. *J. Genet. Genomics*. **41**, 397-407 (2014).

53 Liu, J. *et al.* Deciphering the signature of selective constraints on cancerous mitochondrial genome.*Mol. Biol. Evol.* **29**, 1255-1261 (2012).

54 Derenko, M. *et al.* Phylogeographic Analysis of Mitochondrial DNA in Northern Asian Populations. *Am. J. Hum. Genet.***81**, 1025-1041 (2007).

55 Ji, F. *et al.* Mitochondrial DNA variant associated with Leber hereditary optic neuropathy and high-altitude Tibetans. *Proc. Natl. Acad. Sci.* **109**, 7391-7396 (2012).

56 Gayden, T. *et al.*The Himalayas: Barrier and conduit for gene flow. *Am. J. Phys. Anthropol.*, n/a-n/a (2013).

57 Lee, H. Y. *et al.* East Asian mtDNA haplogroup determination in Koreans: Haplogroup-level coding region SNP analysis and subhaplogroup-level control region sequence analysis. *Electrophoresis* **27**, 4408-4418 (2006).

58 Irwin, J. A. *et al.* Mitochondrial control region sequences from a Vietnamese population sample. *Int. J. Legal Med.* **122**, 257-259 (2008).

59 Summerer M*, et al.* Large-scale mitochondrial DNA analysis in Southeast Asia reveals evolutionary effects of cultural isolation in the multi-ethnic population of Myanmar. *BMC Evol.Biol.* **14,** 17 (2014).

60 Macaulay, V. *et al.* Single, rapid coastal settlement of Asia revealed by analysis of complete mitochondrial genomes. *Science (80-)* **308**, 1034-1036 (2005).

61 Oota, H., Settheetham-Ishida, W., Tiwawech, D., Ishida, T. &Stoneking, M. Human mtDNA and Y-chromosome variation is correlated with matrilocal versus patrilocal residence. *Nat. Genet.* **29**, 20-21 (2001).

62 Peng, M. S. *et al.* Tracing the Austronesian footprint in Mainland Southeast Asia: a perspective from mitochondrial DNA. *Mol. Biol. Evol.* **27**, 2417-2430 (2010).

63 Zimmermann, B. *et al.* Forensic and phylogeographic characterization of mtDNA lineages from northern Thailand (Chiang Mai).*Int. J. Legal Med.* **123**, 495-501 (2009).

64 Black, M., Dufall, K., Wise, C., Sullivan, S. &Bittles, A. Genetic ancestries in northwest Cambodia. *Ann. Hum. Biol.* **33**, 620-627 (2006).

65 Fucharoen, G., Fucharoen, S. &Horai, S. Mitochondrial DNA polymorphisms in Thailand. *J. Hum. Genet.* **46**, 115-125 (2001).

66 Lertrit, P. *et al.* Genetic history of Southeast Asian populations as revealed by ancient and modern human mitochondrial DNA analysis. *Am. J. Phys. Anthropol.* **137**, 425-440, (2008).
67 Hill, C. *et al.* Phylogeography and ethnogenesis of aboriginal Southeast Asians. *Mol. Biol. Evol.* **23**, 2480-2491 (2006).
68 Maruyama, S., Nohira-Koike, C., Minaguchi, K. & Nambiar, P. MtDNA control region sequence polymorphisms and phylogenetic analysis of Malay population living in or around Kuala Lumpur in Malaysia. *Int. J. Legal Med.* **124**, 165-170 (2010).
69 Wong, H. Y. *et al.* Sequence polymorphism of the mitochondrial DNA hypervariable regions I and II in 205 Singapore Malays. *Leg Med* **9**, 33-37 (2007).
70 Haslindawaty, A. R. N., Panneerchelvam, S., Edinur, H. A., Norazmi, M. N. & Zafarina, Z. Sequence polymorphisms of mtDNA HV1, HV2, and HV3 regions in the Malay population of Peninsular Malaysia. *Int. J. Legal Med.* **124**, 415-426 (2010).
71 Bodner, M. *et al.* Southeast Asian diversity: first insights into the complex mtDNA structure of Laos. *BMC Evol. Biol.* **11**, 49 (2011).
72 Roychoudhury, S. *et al.* Genomic structures and population histories of linguistically distinct tribal groups of India. *Hum. Genet.* **109**, 339-350 (2001).
73 Cordaux, R. *et al.* Mitochondrial DNA analysis reveals diverse histories of tribal populations from India. *Eur. J. Hum. Genet.* **11**, 253-264 (2003).
74 Reddy, B. M. *et al.* Austro-Asiatic tribes of Northeast India provide hitherto missing genetic link between South and Southeast Asia. *PLoS ONE* **2**, e1141 (2007).
75 Quintana-Murci, L. *et al.* Where west meets east: the complex mtDNA landscape of the southwest and Central Asian corridor. *Am. J. Hum. Genet.* **74**, 827-845 (2004).
76 Metspalu, M. *et al.* Most of the extant mtDNA boundaries in south and southwest Asia were likely shaped during the initial settlement of Eurasia by anatomically modern humans. *BMC Genet.* **5**, 26 (2004).
77 Sharma, S., Saha, A., Rai, E., Bhat, A. & Bamezai, R. Human mtDNA hypervariable regions, HVR I and II, hint at deep common maternal founder and subsequent maternal gene flow in Indian population groups. *J. Hum. Genet.* **50**, 497-506 (2005).
78 Mountain, J. L. *et al.* Demographic history of India and mtDNA-sequence diversity. *Am. J. Hum. Genet.* **56**, 979-992 (1995).
79 Thangaraj, K. *et al.* Different population histories of the Mundari-and Mon-Khmer-speaking Austro-Asiatic tribes inferred from the mtDNA 9-bp deletion/insertion polymorphism in Indian populations. *Hum. Genet.* **116**, 507-517 (2005).
80 Barnabas, S., Shouche, Y. & Suresh, C. High-Resolution mtDNA Studies of the Indian Population: Implications for Palaeolithic Settlement of the Indian Subcontinent. *Ann. Hum. Genet.* **70**, 42-58 (2006).
81 Rajkumar, R. & Kashyap, V. Haplotype diversity in mitochondrial DNA hypervariable regions I and II in three communities of Southern India. *Forensic Sci. Int.* **136**, 79-82 (2003).
82 Rajkumar, R. & Kashyap, V. Mitochondrial DNA hypervariable region I and II sequence polymorphism in the Dravidian linguistic group of India. *J. Forensic Sci.* **48**, 227 (2003).
83 Thangaraj, K., Ramana, G. V. & Singh, L. Y-chromosome and mitochondrial DNA polymorphisms in Indian populations. *Electrophoresis* **20**, 1743-1747 (1999).
84 Kivisild, T. *et al.* Deep common ancestry of Indian and western-Eurasian mitochondrial DNA lineages. *Curr. Biol.* **9**, 1331-1334 (1999).

85 Quintana-Murci, L. *et al.* Genetic evidence of an early exit of Homo sapiens sapiens from Africa through eastern Africa. *Nat. Genet.* **23**, 437-441 (1999).
86 Thanseem, I. *et al.* Genetic affinities among the lower castes and tribal groups of India: inference from Y chromosome and mitochondrial DNA. *BMC Genet.* **7**, 42 (2006).
87 Kumar, V. *et al.* Global patterns in human mitochondrial DNA and Y-chromosome variation caused by spatial instability of the local cultural processes. *PLoS Genet.* **2**, e53 (2006).
88 Kivisild, T. *et al.* The genetic heritage of the earliest settlers persists both in Indian tribal and caste populations. *Am. J. Hum. Genet.* **72**, 313-332 (2003).
89 Watkins, W. *et al.* Multiple origins of the mtDNA 9-bp deletion in populations of South India. *Am. J. Phys. Anthropol.* **109**, 147-158 (1999).
90 Mittal, B. *et al.* Mitochondrial DNA variation and substructure among the tribal populations of Andhra Pradesh, India. *Am. J. Hum. Biol.* **20**, 683-692 (2008).
91 Gaikwad, S. & Kashyap, V. Molecular insight into the genesis of ranked caste populations of western India based upon polymorphisms across non-recombinant and recombinant regions in genome. *Genome Biol.* **6**, P10 (2005).
92 Roy, S., Thakur, C. & Majumder, P. P. Mitochondrial DNA variation in ranked caste groups of Maharashtra (India) and its implication on genetic relationships and origins. *Ann. Hum. Biol.* **30**, 443-454 (2003).
93 Baig, M., Khan, A. & Kulkarni, K. Mitochondrial DNA diversity in tribal and caste groups of Maharashtra (India) and its implication on their genetic origins. *Ann. Hum. Genet.* **68**, 453-460 (2004).
94 Kaur, I. *et al.* Genomic diversities and affinities among four endogamous groups of Punjab (India) based on autosomal and mitochondrial DNA polymorphisms. *Hum. Biol.*, 819-836 (2002).
95 Banerjee, J., Trivedi, R. & Kashyap, V. Mitochondrial DNA control region sequence polymorphism in four indigenous tribes of Chotanagpur plateau, India. *Forensic Sci. Int.* **149**, 271-274 (2005).
96 Sahoo, S. & Kashyap, V. Phylogeography of mitochondrial DNA and Y-Chromosome haplogroups reveal asymmetric gene flow in populations of Eastern India. *Am. J. Phys. Anthropol.* **131**, 84-97 (2006).
97 Thangaraj, K. *et al.* Genetic affinities of the Andaman Islanders, a vanishing human population. *Curr. Biol.* **13**, 86-93 (2003).
98 Ravi Prasad, B. *et al.* Mitochondrial DNA variation in Nicobarese islanders. *Hum. Biol.* **73**, 715-725 (2001).
99 Wirth, T. *et al.* Distinguishing human ethnic groups by means of sequences from Helicobacter pylori: lessons from Ladakh. *Proc. Natl. Acad. Sci.* **101**, 4746-4751 (2004).
100 Eaaswarkhanth, M. *et al.* Traces of sub-Saharan and Middle Eastern lineages in Indian Muslim populations. *Eur. J. Hum. Genet.* **18**, 354-363 (2009).
101 Fornarino, S. *et al.* Mitochondrial and Y-chromosome diversity of the Tharus (Nepal): a reservoir of genetic variation. *BMC Evol. Biol.* **9**, 154 (2009).
102 Malyarchuk, B., Derenko, M., Denisova, G. & Kravtsova, O. Mitogenomic diversity in Tatars from the Volga-Ural region of Russia. *Mol. Biol. Evol.* **27**, 2220-2226 (2010).
103 Kolman, C. J., Sambuughin, N. & Bermingham, E. Mitochondrial DNA analysis of Mongolian populations and implications for the origin of New World founders. *Genetics* **142**,

1321-1334 (1996).

104 Keyser-Tracqui, C., Crubezy, E., Pamzsav, H., Varga, T. & Ludes, B. Population origins in Mongolia: genetic structure analysis of ancient and modern DNA. *Am. J. Phys. Anthropol.* **131**, 272-281 (2006).

105 Derenko, M. V. *et al.* Mitochondrial DNA variation in two South Siberian Aboriginal populations: implications for the genetic history of North Asia. *Hum. Biol.*, 945-973 (2000).

106 Malyarchuk, B. & Derenko, M. Mitochondrial DNA variability in Russians and Ukrainians: Implication to the origin of the Eastern Slavs. *Ann. Hum. Genet.* **65**, 63-78 (2001).

107 Derenko, M. *et al.* Diversity of mitochondrial DNA lineages in South Siberia. *Ann. Hum. Genet.* **67**, 391-411 (2003).

108 Pakendorf, B. *et al.* Mitochondrial DNA evidence for admixed origins of central Siberian populations. *Am. J. Phys. Anthropol.* **120**, 211-224 (2003).

109 Fedorova, S., Bermisheva, M., Villems, R., Maksimova, N. & Khusnutdinova, E. Analysis of mitochondrial DNA haplotypes in yakut population]. *Mol. Biol. (Mosk.)* **37**, 643 (2003).

110 Derenko, M. & Shields, G. Diversity of mitochondrial DNA nucleotide sequences in three groups of aboriginal inhabitants of Northern Asia]. *Mol. Biol. (Mosk.)* **31**, 784 (1997).

111 Derbeneva, O., Starikovskaia, E., Volod'ko, N., Wallace, D. & Sukernik, R. [Mitochondrial DNA variation in Kets and Nganasans and the early peoples of Northern Eurasia]. *Genetika* **38**, 1554-1560 (2002).

112 Bermisheva, M., Tambets, K., Villems, R. & Khusnutdinova, E. [Diversity of mitochondrial DNA haplotypes in ethnic populations of the Volga-Ural region of Russia]. *Mol. Biol. (Mosk.)* **36**, 990-1001 (2001).

113 Starikovskaya, E. B. *et al.* Mitochondrial DNA diversity in indigenous populations of the southern extent of Siberia, and the origins of Native American haplogroups. *Ann. Hum. Genet.* **69**, 67-89 (2005).

114 Volodko, N. V. *et al.* Mitochondrial genome diversity in arctic Siberians, with particular reference to the evolutionary history of Beringia and Pleistocenic peopling of the Americas. *Am. J. Hum. Genet.* **82**, 1084-1100 (2008).

115 Dulik, Matthew C. *et al.* Mitochondrial DNA and Y Chromosome Variation Provides Evidence for a Recent Common Ancestry between Native Americans and Indigenous Altaians. *Am. J. Hum. Genet.* **90**, 229-246 (2012).

116 Derenko, M. *et al.* Complete mitochondrial DNA analysis of eastern Eurasian haplogroups rarely found in populations of northern Asia and eastern Europe. *PLoS ONE* **7**, e32179 (2012).

117 Pimenoff, V. N. *et al.* Northwest Siberian Khanty and Mansi in the junction of West and East Eurasian gene pools as revealed by uniparental markers. *Eur. J. Hum. Genet.* **16**, 1254-1264 (2008).

118 Derenko, M. *et al.* Origin and post-glacial dispersal of mitochondrial DNA haplogroups C and D in northern Asia. *PLoS ONE* **5**, e15214 (2010).

119 Sukernik, R. I. *et al.* Mitochondrial genome diversity in the tubalar, even, and ulchi: Contribution to prehistory of native siberians and their affinities to native americans. *Am. J. Phys. Anthropol.* **148**, 123-138 (2012).

120 Comas, D. *et al.* Admixture, migrations, and dispersals in Central Asia: evidence from maternal DNA lineages. *Eur. J. Hum. Genet.* **12**, 495-504 (2004).

121 Irwin, J. A. *et al.* The mtDNA composition of Uzbekistan: a microcosm of Central Asian

patterns. *Int. J. Legal Med.* **124**, 195-204 (2010).
122 Chaix, R. *et al.* From social to genetic structures in central Asia. *Curr. Biol.* **17**, 43-48 (2007).
123 Lalueza-Fox, C. *et al.* Unravelling migrations in the steppe: mitochondrial DNA sequences from ancient Central Asians. *Proc. R. Soc. Lond., B, Biol. Sci.* **271**, 941-948 (2004).
124 Al-Zahery, N. *et al.* Characterization of mitochondrial DNA control region lineages in Iraq. *Int. J. Legal Med.* **127**, 373-375 (2013).
125 Scheible, M. *et al.* Mitochondrial DNA control region variation in a Kuwaiti population sample. *Forensic Sci Int Genet* **5**, e112-e113 (2011).
126 Cardoso, S. *et al.* The maternal legacy of Basques in northern navarre: New insights into the mitochondrial DNA diversity of the Franco-Cantabrian area. *Am. J. Phys. Anthropol.* **145**, 480-488 (2011).
127 Cardoso, S. *et al.* The Expanded mtDNA Phylogeny of the Franco-Cantabrian Region Upholds the Pre-Neolithic Genetic Substrate of Basques. *PLoS ONE* **8**, e67835 (2013).
128 Álvarez-Cubero, M. J. *et al.* Mitochondrial Haplogroups and Polymorphisms Reveal No Association with Sporadic Prostate Cancer in a Southern European Population. *PLoS ONE* **7**, e41201 (2012).
129 Bertolin, C. *et al.* Analysis of complete mitochondrial genomes of patients with schizophrenia and bipolar disorder. *J. Hum. Genet.* **56**, 869-872 (2011).
130 Achilli, A. *et al.* Mitochondrial DNA backgrounds might modulate diabetes complications rather than T2DM as a whole. *PLoS ONE* **6**, e21029 (2011).
131 Pierron, D. *et al.* New evidence of a mitochondrial genetic background paradox: impact of the J haplogroup on the A3243G mutation. *BMC Med. Genet.* **9**, 41 (2008).
132 Mogentale-Profizi, N. *et al.* Mitochondrial DNA sequence diversity in two groups of Italian Veneto speakers from Veneto. *Ann. Hum. Genet.* **65**, 153-166 (2001).
133 Cardoso, S. *et al.* Variability of the entire mitochondrial DNA control region in a human isolate from the Pas Valley (northern Spain). *J. Forensic Sci.* **55**, 1196-1201 (2010).
134 Achilli, A. *et al.* Mitochondrial DNA variation of modern Tuscans supports the near eastern origin of Etruscans. *Am. J. Hum. Genet.* **80**, 759-768 (2007).
135 Irwin, J. *et al.* Mitochondrial control region sequences from northern Greece and Greek Cypriots. *Int. J. Legal Med.* **122**, 87-89, (2008).
136 Di Benedetto, G. *et al.* Mitochondrial DNA sequences in prehistoric human remains from the Alps. *Eur. J. Hum. Genet.* **8**, 669-677 (2000).
137 Bogacsi-Szabo, E. *et al.* Mitochondrial DNA of ancient Cumanians: culturally Asian steppe nomadic immigrants with substantially more western Eurasian mitochondrial DNA lineages. *Hum. Biol.*, 639-662 (2005).
138 Helgason, A. *et al.* mtDNA and the islands of the North Atlantic: estimating the proportions of Norse and Gaelic ancestry. *Am. J. Hum. Genet.* **68**, 723-737 (2001).
139 Malyarchuk, B. *et al.* Mitochondrial DNA variability in Poles and Russians. *Ann. Hum. Genet.* **66**, 261-283 (2002).
